# Disrespectful care in family planning services among youth and adult simulated clients in public sector facilities in Malawi

**DOI:** 10.1186/s12913-021-06353-z

**Published:** 2021-04-14

**Authors:** Elizabeth Hazel, Diwakar Mohan, Ephraim Chirwa, Mary Phiri, Fannie Kachale, Patrick Msukwa, Joanne Katz, Melissa A. Marx

**Affiliations:** 1 Johns Hopkins Bloomberg School of Public Health, Baltimore, USA; 2 Wadonda Consult Limited, Zomba, Malawi; 3 Ministry of Health, Malawi, Lilongwe, Malawi

**Keywords:** Family planning, Respectful care, Quality of care

## Abstract

**Background:**

Provision of high-quality family planning (FP) services improves access to contraceptives. Negative experiences in maternal health have been documented worldwide and likely occur in other services including FP. This study aims to quantify disrespectful care for adult and adolescent women accessing FP in Malawi.

**Methods:**

We used simulated clients (SCs) to measure disrespectful care in a census of public facilities in six districts of Malawi in 2018. SCs visited one provider in each of the 112 facilities: two SCs visits (one adult and one adolescent case scenario) or 224 SC visits total. We measured disrespectful care using a quantitative tool and field notes and report the prevalence and 95% confidence intervals for the indicators and by SC case scenarios contextualized with quotes from the field notes.

**Results:**

Some SCs (12%) were refused care mostly because they did not agree to receive a HIV test or vaccination, or less commonly because the clinic was closed during operating hours. Over half (59%) of the visits did not have privacy. The SCs were not asked their contraceptive preference in 57% of the visits, 28% reported they were not greeted respectfully, and 20% reported interruptions. In 18% of the visits the SCs reported humiliation such as verbal abuse. Adults SCs received poorer counseling compared to the adolescent SCs with no other differences found.

**Conclusions:**

We documented instances of refusal of care, lack of privacy, poor client centered care and humiliating treatment by providers. We recommend continued effort to improve quality of care with an emphasis on client treatment, regular quality assessments that include measurement of disrespectful care, and more research on practices to reduce it.

**Supplementary Information:**

The online version contains supplementary material available at 10.1186/s12913-021-06353-z.

## Background

Contraceptive use has increased dramatically worldwide in the last 50 years but unmet need for family planning is unacceptably high [1]. In 2017, 208 million women living in low-and-middle income countries (LMICs) wanted to limit births but were not using modern contraceptives, and these women accounted for 84% of unintended births globally [2]. The gap in contraceptive prevalence rates between the wealthiest and the poorest women has been decreasing in LMICs but has remained stagnant or is increasing in sub-Saharan Africa [3]. Not only does access to high quality family planning services reduce unintended pregnancies but is a human right as reaffirmed at the 1994 International Conference on Population and Development, and in the global strategy to extend universal access to sexual and reproductive health care, as defined by the Sustainable Development Goals [4–7].

A framework for quality family planning care was first defined by Judith Bruce’s seminal paper [8]. She defined quality family planning services as technical competency, or how well the provider adheres to clinical guidelines and best practices, and also included a positive interpersonal relationship between provider and client, a supportive environment for client choice of contraceptive methods, and respectful, dignified care [8]. More recent work has expanded the importance of client-centered, respectful care and further grounded quality of care in human rights for family planning [9, 10]. Research shows women who have agency over their method choice, are satisfied with their services, and are treated respectfully are more likely to have their contraceptive needs met. A literature summary by RamaRao & Mohanam describes several studies showing a positive association of client-perceived quality of care with contraceptive adoption/continuation in Niger, Bangladesh, Tanzania, and The Gambia [11]. Abdel-Tawab & RamaRao summarized studies in LMICs showing a positive association of provider-client interactions on contraceptive continuation but found limited evidence that interventions to improve quality of interactions impacted continuation [12]. They theorized this lack of consistency is related to differences in methodological design across the studies, but also the general complexity of both provider-client interactions and contraceptive continuation. A more recent literature review of studies in LMICs and high income countries by Diamond-Smith et al., shows interventions aimed to support client dignity, autonomy, privacy/confidentiality, and communication are associated with improved client satisfaction and knowledge, but there are mixed associations/findings with contraceptive initiation and continuation [13].

However, there is little information available on the prevalence of negative treatment by family planning providers and how it may impact client satisfaction, knowledge, or contraceptive use. Negative client experiences in maternal health and delivery are documented globally - in both LMICs and high income countries - including verbal or physical abuse, lack of privacy, non-consented care, and discrimination [14, 15]. One third of patients experienced mistreatment during labor and delivery in a cross-sectional observation study and survey of women in Ghana, Guinea, Myanmar, and Nigeria, showing pervasiveness of the problem [16]. Disrespect and abuse of women accessing family planning care has been documented in LMICs in previous decades but has not been the primary focus of the studies and thus reports have been anecdotal. Schuler et al. in 1985 Nepal found that simulated clients from a lower caste received poorer counseling and less respectful care during family planning services compared to SCs from higher castes [17]. A 1995 study in Senegal found the SC youth (less than 20 years of age) accessing FP services in seven facilities in Dakar region had difficulty accessing the providers and experienced unwelcoming attitudes once at the facility [18]. Nalwadda et al. conducted a study in public and private sector facilities in Uganda in 2010 using youth-aged (15–25 years of age) SCs accessing FP services and found providers raised their voice or shouted at the simulated clients in 4% of the interactions [19]. Adult simulated clients (aged 23–30) from a 2012 study in 19 public and private sector facilities in Kenya reported instances of negative comments or rude behavior by family planning providers [20].

Reporting on instances of disrespectful care indicates the magnitude of the issue and can help identify policy, facility, and provider level issues to target for program improvement. As previously described, women who are satisfied with their care and experience positive provider interactions are more likely to initiate/continue contraceptive use, and interventions to improve respectful care lead to this increased satisfaction and knowledge. This study aims to document and quantify any instances of disrespectful care for adult and adolescent women accessing family planning through a cross-sectional facility assessment in Malawi.

Malawi is a low-income country in southern Africa with a population of 17 million in 2018, expected to double by 2042 [21]. According to the 2015–2016 Malawi Demographic and Health Survey (DHS), the proportion of married women with unmet need in Malawi has declined from nearly 40% in the mid-1990s to 19% in 2016, but there is wide variation sub-nationally, ranging from 30 to 12% by district [22]. Women 15–19 years old have lower demand satisfied for family planning services (62%) compared to women 20 years and older (75% or higher) [22]. Only a third of sexually active unmarried women between 15 and 19 years of age are using modern contraceptives [22].

The 2009 Malawi National Sexual and Reproductive Health and Rights policy committed to a human rights-based delivery approach and providing health services to all [23, 24]. Contraceptive services are free in Malawi through the government program financed by donor institutions, and 80% of all women and 77% of women between 15 and 19 years of age using modern contraceptives get them from the public sector [22, 25]. The method mix in Malawi in skewed, most married/in union women using a modern contraceptive are using injectable hormonal contraceptives (52%) followed by implants (20%), and female sterilization (19%) [22]. Services are delivered through hospitals, health centers, dispensaries, clinics and health posts, and 95% of all government-managed facilities offer at least one modern contraceptive method [26]. Family planning providers are trained to counsel on all sexual reproductive health services provided, describe those available at the facility (refer if necessary), guide the client in making an informed decision, and be respectful and communicative [27].

## Methodology

### Sample

This sub-study was part of a larger evaluation of the national family planning program in Malawi [28]. The aim of the evaluation was to determine whether district-level differences in fertility and contraceptive use were associated with quality of family planning services. The evaluation measured quality of care using direct observation, client exit interviews, knowledge assessments, and simulated clients (SCs). SCs are trained to act as clients seeking services in order to evaluate care without the provider knowing that they were being assessed [29]. This sub-study used the SC protocol and expanded it to collect information related to disrespectful care for facility-based family planning providers.

For the larger evaluation, we selected six of the 28 administrative districts in Malawi: one group of three low FP outcome districts and another group of three FP high outcome districts. We developed a district database of FP outcomes from the 2015–2016 DHS: changes in total fertility rate, modern contraceptive prevalence, unmet need, demand for FP services satisfied, and adolescent pregnancy. We purposefully selected Chitipa, Dedza and Salima as the low outcome group based on the FP outcomes relative to the other districts, and to maximize variation in geographic spread and religious representation (i.e. at least one district had a significant Muslim population). We matched this district group to high outcome districts (Machinga, Mangochi and Nkhata Bay) based on theorized confounders including proportion of rural households, women’s education, religion, poverty, and facilities per population by district using coarsened exact matching – method of matching by categories of values rather than exact values [30]. This study is a census of all public sector facilities in the six districts. We did not include private for-profit facilities, facilities managed by non-government organization (e.g. Banja La Mtsogolo), or religious facilities that do not offer contraceptives.

### Tool development

In 2016, Harris et al. published a specific framework of disrespectful care and abuse for family planning by applying a framework previously developed for intrapartum care [31]. They defined respectful family planning care as support for women’s contraceptive method choice free from coercion, and creation of a positive, client-centered environment [31]. Adapting this framework, we defined four domains of disrespectful care: poor client-centered care, non-private consultations, refusal of care, and non-dignified care [31]. We conducted a desk review of existing quality of care indicators and assessment protocols that measured provider-delivered family planning care [26, 32–38].^1^ For each domain of the framework, we identified measurable indicators given the study design, used the questions from existing tools where they existed and developed new questions if needed (Table 1). For instance, for the poor client-center care domain, one indicator we measured was the proportion of visits where the provider did not ask the client preferred method. For privacy, we measured the proportion of visits where there was not auditory or visual privacy. We developed the non-dignified care questions based on reported provider behaviors from a qualitative study on adolescent perceptions of family planning in Malawi [39].

**Table 1:** Questions used to develop indicators of disrespectful care

Construct	Indicator	Questions from qualitative tool used to create indicator
**Domain: Refusal of care**		
Refusal of services due to non-essential procedures	% of visits where the SCs did not receive method due to refusal of HIV test, TT vaccination or clinic was closed when it was supposed to be open	• Were you prescribed, given or referred for a method during this visit? • If no, referred to field notes for reason.
**Domain: Non-private consultation**		
Client services are not provided with visual or auditory privacy	% of visits that took place in a group setting, or without visual and auditory privacy	• Did the provider talk to you about your family planning methods in a group or by yourself? • If individual counseling, was the consultation conducted in an area where no one could see you and provider? • If individual counseling, was the consultation conducted in an area where no one could hear your conversation?
**Domain: Poor client-centered care**		
Incomplete family planning options given by provider(s)	% of visits where the provider did not mention any methods beyond what the SC requested (pills)	• Did the provider talk to you (or the group) about any contraceptive methods? • If yes, what contraceptive method(s) did the provider talk with you about?
Inaccurate information by provider(s)	Not measured through quantitative tool.	
Client unsupported in personal method choice	% of visits where the provider advocated for a method	• Did the provider talk to you (or the group) about any contraceptive methods? • If yes, do you feel the provider advocated a specific method for you during the consultation?
	% of visits where the provider did not ask client preference	• Did the provider talk to you (or the group) about any contraceptive methods? • If yes, did the provider ask you about your preference in contraceptive methods?
Poor listening and attention by provider(s)	% of visits where the provider did not greet SCs (or the group) respectfully	• Did the provider greet you (or the group) respectfully?
	% of visits where the provider interrupted the SCs while speaking or interrupted consultation for other business	• Did the provider interrupt you while you were speaking? • Did the provider interrupt the consultation to conduct other business?
**Domain: Non-dignified care**		
Clients experience humiliating treatment from providers or other health staff	% of visits where the provider SCs experienced humiliating treatment such as yelling, threatening, scolding, or being insulted	• Did provider raise their voice or yell at you? • Did provider use a disparaging term to describe you? • Did provider do anything else considered disrespectful or abusive? • If yes, what did the provider say or do? • Did the provider make any critical or judgmental comments about: • The number of children you have? Or do not have? • Your plans for whether you want to have more children and when? • Your partner/marital status? • The involvement of your partner in your family planning? • Your sexual activity? • The involvement of your parents? • Your age in regards to accessing family planning? • Your preferred method of contraceptives? • Your physical appearance? Judgement comments by staff • At any point, did you feel unwelcome by other health facility staff? • At any point, did other health facility staff make disrespectful or judgmental remarks to you or about you to others where you could overhear? • If yes, what did the staff say or do?
Clinic level barriers to care	% of visits where the SCs waited longer than one hour for services	• How long did you wait to be seen?
	% of visits where the provider asked for additional money (informal payments)	• Did provider ask for additional money (informal payment)? • Did any other health facility staff ask you for additional money (informal payment)?

We then created a quantitative tool with pre-coded responses and translated it into Chichewa and Tumbuka, two languages spoken in Malawi. During the early stages of data collection, it was apparent the details of the SC encounters with the health system could not be adequately captured in a pre-coded, quantitative tool. To capture this, we added an ad hoc qualitative field notes tool or a notebook for the SCs to describe their encounters at the facility and with the providers.

The case scenarios were adapted from Malawi-specific family planning training materials, pretested with non-study clinicians in Malawi, and reviewed by a SC training consultant working with a Malawian organization for clinical and cultural accuracy (Additional file 1) [40–42]. One scenario was a married, adult, “method-switcher” who wants to change from hormonal injectable contraceptives to hormonal contraceptive pills. The other was an adolescent, unmarried woman who has just become sexually active and is a “first-time user” of contraceptives. To elicit comparable care across the providers, the details of each scenario were standardized including medical history, parity, age, and method preference among other factors (Additional file 1).

### Data collection

The study team hired twelve data collectors with previous survey experience for the simulated clients: six assigned to an adult case scenario and six assigned to an adolescent case scenario. The SCs participated in a two-week training for the main study, including a one-day training focused entirely on client simulation, and a pilot to practice their scenario at a non-study facility. The survey coordination team assigned six data collectors to the adolescent SC based on their resemblance to younger woman; all data collectors were over 18 years of age.

For the medical safety of the data collectors, the SCs could only accept pills or condoms and were trained to deploy standardized exit strategies to avoid injectables, implants, IUDs, or other invasive procedures (e.g. needle stick or cervical exam). To avoid invasive procedures, the SCs were trained to tell the provider they would come back at a later date for the procedure and ask for pills or condoms to use in the meantime. We selected hormonal contraceptive pills as the method preference since the counseling is more complex compared to condoms and measuring counseling quality was one of the aims of the overall study. SCs were trained to provide the information from their assigned scenario when asked by the provider to mimic an actual consultation.

Data collection was carried out from January through March 2018. There were six teams, one team assigned to a district and two SCs (adult and adolescent SC) per team. SCs visited the first facility in each district approximately 1 to 2 days after all the listed providers in that district were consented, and the last SC visit occurred 61 days later. Each facility was visited once by two SCs. Both the “Adult” and “Adolescent” SCs arrived at a facility on the same morning, traveling separately. All SCs presented as clients accessing the facility for the first time.

When the consultation was complete, the SCs returned to the field vehicle parked out sight of the facility and were immediately interviewed by their supervisor in the vehicle (unobserved by community members) using the quantitative tool. The supervisor then returned to interview the providers who consulted the SCs with a standardized instrument that solicited their background characteristics, education, and information about their position. Later that evening, the SCs recorded their encounter in the field notes.

All providers gave their consent to participate in the study. One team inadvertently deleted a SC form from the data collection device before transfer to the server. In this study, we included data from 111 facilities (> 99% response rate) or 222 SC consultations (111 adult and 111 adolescent consultations).

### Recruitment and consent process

We created a listing of public sector facilities that offer family planning services in the six districts by updating a 2017 census of family planning facilities through discussions with the District Health Office [28]. Study teams contacted each of the facility administrators to create a listing of all family planning providers working at that facility. From these listings, all facility-based providers in the six districts were called prior to data collection for a verbal consent using a standardized form. The consent form stated the providers may be visited by masked, simulated clients sometime in the next 3 months. The provider offering family planning services the day the SCs visited the facility were included in the study. If two providers worked as a team the more senior provider was enrolled.

During the provider interviews conducted after the SC consultations, the supervisors confirmed whether the provider had given consent to participation in the study. If not, the study team supervisor read the consent form to the provider and they were given the opportunity to be removed from the study and their data deleted. Reasons for not initially being consented by mobile phone include poor network connectivity, new hire or transfer from another facility, or the provider was missed when the facility in-charge listed the family planning providers.

### Data management and analysis

We entered the checklist responses into Open Data Kit on Android tablets and used R and Stata 14.2 software for analysis [43–45]. .We reported the prevalence of disrespectful actions and associated 95% confidence intervals for the six districts aggregated, assuming a binomial distribution with no survey design effect. We conducted a stratified analysis to determine whether the indicators differed by district, case scenario or phase of data collection. Since many of the indicators were subjective, we aimed to test whether SC reporting changed from the first half (< 30 days) versus the second half (> 30 days) of data collection. To compare the levels of care for these stratified analyses, we reported the proportions and compared 95% confidence intervals among the groups.

We digitized the field notes from the SCs for analysis in Microsoft Excel and they were coded and organized by theme using a framework analysis as described by Ritchie and Lewis [46, 47]. If we found an event that fit within the four domains of disrespectful care, it was coded with a binary score and triangulated with the quantitative data. We selected excerpts from the translated field notes to provide descriptive context to the quantitative data in each domain.

## Results

### Facility and provider characteristics

Equal proportions of providers were female and male and 40% were under the age of 30 years of age (Table 2). Most providers were married (75%) and Protestant Christian (71%). Over half (64%) were nurses and 27% were community health providers known as Health Surveillance Assistants (HSAs), who also can provide services in facilities. Mangochi district had the greatest number of facilities, and the greatest number of providers (23%) (Table 2). Generally, there is one hospital per district and the remaining are clinics or dispensaries (data on facility type not collected).

**Table 2:** Characteristics of facility-based providers with complete data who provided care to the simulated clients

Provider characteristics	% (***n*** = 111)
**District**	
*Chitipa*	9
*Dedza*	22
*Machinga*	15
*Mangochi*	23
*NkhataBay*	19
*Salima*	12
**Provider gender**	
*Female*	51
*Male*	49
**Provider age**	
*20–29*	41
*30–39*	29
*40+*	31
**Provider marital status**	
*Married (traditional, religious, or civil marriage)*	75
*In a relationship, but not married*	20
*Single*	5
**Provider religion**	
*Catholic Christian*	14
*Muslim*	12
*Protestant Christian*	71
*Other*	3
**Provider title**	
*Medical assistant*	9
*Registered Nurse or midwife*	3
*Enrolled Nurse or midwife*	41
*Community Nurse or midwife*	20
*Health Surveillance Assistants (HSAs)*	27
**Years at position**	
*0–1 years*	22
2–5 years	35
6–10 years	12
*> 10 years*	32
**Provider education**	
*Primary or Secondary certificate*	11
*Malawi School Certificate of Education*	16
*College Certificate*	29
*College Diploma*	42
*Refused to answer*	2

### Findings by domain

#### Refusal of care

We considered two reasons for refusal of care: the facility was closed on reported days of operations or the facility mandated HIV testing and counseling (HTC) and/or tetanus toxoid vaccination (TTV) to receive family planning services. Almost 12% of the simulated clients did not receive their preferred method. In over half of these visits, the SCs did not receive it due to refusal of HTC (7%) and a smaller percentage reported it due to refusal of TTV (2%), refusal of both HTC and TTV (1%) or facility closure (2%) (data not shown).

*“Provider told me to go for HTC before I can access services. Provider said it is compulsory for me to do the test. If I refused then they will not assist me. So I told the provider that I was not ready for the test hence I’m going home. Provider told me to go only come again when I’m ready for the test. So I exited that facility without any method.”*

– Adolescent SC

#### Non-private care

We found facilities conducted counseling with individual clients or multiple clients at the same time (group counseling). Over half the visits (59%) took place in a group setting or individually with no visual or auditory privacy (Table 3). Over half of the counseling sessions (54%) were held as a group. Out of the individual consultations (*n* = 90), 76% had both visual and auditory privacy.

*“The family planning room had no visual privacy and we were more than 6 women in room to get family planning methods at once.” –* Adolescent SC

**Table 3:** Proportion of consultations with disrespectful care total, and by adult and adolescent simulated clients (SCs)

Constructs	Indicators	Total % (95% CI) *n* = 222	Adult SC % (95% CI) *n* = 111	Adol. SC % (95% CI) *n* = 111
**Refusal of care**				
Refusal of services	% of consultations where the SCs did not receive method due to refusal of HIV test, TT vaccination or clinic was closed when it was supposed to be open	12 (8, 17)	12 (7, 19)	12 (7, 19)
**Non-private consultations**				
Client services are not provided with visual or auditory privacy	% of consultations that took place in a group setting, or without visual and auditory privacy	59 (52, 67) *N* = 167^a^	56 (45, 66) *N* = 81^a^	63 (52, 72) *N* = 86^a^
**Poor client-centered care**				
Incomplete family planning options given by provider(s)	% of consultations where the provider did not mention any methods beyond what the SC requested (pills)	56 (49, 62)	69 (60, 77)	42 (34, 52)
Client unsupported in personal method choice	% of consultations where the provider advocated for a method during consultation	5 (3, 9)	4 (1, 9)	7 (4, 14)
	% of consultations where the provider did not ask client preference	57 (50, 63)	69 (59, 77)	45 (36, 55)
Poor listening and attention by provider(s)	% of consultations where the provider did not greet SCs (or the group) respectfully	28 (22, 34)	31 (23, 40)	24 (17, 33)
	% of consultations where the provider interrupted the SCs while speaking or interrupted consultation for other business	20 (15, 26)	22 (15, 30)	18 (12, 26)
**Non-dignified care**				
Clients experience humiliating treatment from providers or other health staff	% of consultations where the provider SCs experienced humiliating treatment such as yelling, threatening, scolding, or being insulted	18 (13, 23)	18 (12, 26)	17 (11, 25)
	*Provider or other health staff raised voice/yelled at SCs*	6 (4, 10)	8 (4, 15)	5 (2, 11)
	*Provider or other health staff made judgmental comments about SC young age and use of family planning services*	NA	0	5 (2, 12)
	*Provider or other health staff expressed anger with the SCs refusal to comply with clinic procedures*	5 (3, 9)	5 (2, 12)	5 (2, 11)
Clinic-level barriers to care	% of consultations where the SCs waited longer than one hour for services	29 (23, 35)	25 (18, 34)	32 (24, 42)
	% of consultations where the provider asked for additional money	0	0	0
Total	% of consultations where at least one of the above occurred	95 (92, 97)	97 (92, 99)	93 (86, 96)

^a^Excludes those with no counseling, *NA* Not applicable

#### Poor client-centered care

The SCs were not asked their preference for family planning method in 57% of the visits (Table 3). In 5% of the visits, the SCs documented that they felt the provider was advocating for a specific method. In over half the visits (56%), the provider did not mention any additional methods besides hormonal pills, which was the SC’s stated preferred method (Table 3).

*“We were not asked our preferred method but they assumed that we all came for injectable. I had to tell the provider that I wanted pills.”* – Adolescent SC

We did not collect information on counseling inaccuracies, however in the field notes the SC recorded two events where they were counseled with inaccurate information on injectables and implants.

*“One of the HSA who was providing the contraceptive injectable told me that I am too young to access methods. Methods will destroy my bones.”*

- Adolescent SC

*“During group counselling the provider (HSA) warned us ‘don’t get tempted to use some of these satanic family planning methods like the implant. Whites are clever they always want to try out things on us blacks and Asians. Some of these are not good. They will just drain your blood’.”*

- Adult SC

In 28% of the visits, SCs reported they were not greeted respectfully and in 20% of the visits, the SCs reported that the provider either interrupted them while they were speaking or interrupted the consultation to conduct unrelated business (Table 3). For example, during a visit one adolescent SC reported the nurse was using *Whatsapp* on their phone throughout the visit.

#### Non-dignified care

In 18% of the visits the SC reported humiliating treatment (Table 3). In 6% of the visits the provider or staff person yelled at the client. Some providers (5% overall) raised their voice or yelled at the SCs after they declined to consent to HTC and/or TTV.

*“[Provider] stationed at the Health Centre forced me to go for an HIV test, I refused. She raised her voice at me for refusing to get tested.* – Adult SC

The adolescent SCs reported judgmental treatment related to their (simulated) age and marital status in 5% of the visits.

*“*[Provider] *counseled me to abstain not trusting my boyfriend in order to finish school properly. I was given pills and condoms for backup if my boyfriend insists to have sex before 7 days and the provider said that am young and should not be thinking of relationships.”* – Adolescent SC

In one case out of the 222 consultations, the SC documented verbal sexual harassment by a family planning provider in the field notes. Using our framework, we defined this one documented instance as “Non-dignified care”. However, all the adolescent SCs reported to their team supervisors or the field coordinator being asked for their phone numbers by health providers seeking further relationship at one or more of the study facilities, although they did not document this in the field notes.^2^

*“One of the health surveillance assistant who was also assisting* [family planning] *clients was proposing me for a relationship.* [He was saying … ] *‘Give me your number. Let us meet somewhere away from the facility for where we can discuss. Where do you live? Please, be serious. I am serious. You can flash me on this number’”* – Adolescent SC

The median waiting times for the SCs was 1 h, ranging from immediately being seen to waiting 4 h. Nearly a third of the SCs reported waiting longer than 1 h for services (29%) (Table 3). None of the SCs documented being asked for additional payments but in the field notes they recorded two events that may be related to additional payments.

*“The provider told us in a group that everyone should be menstruating of which he was to check to confirm, if* [not menstruating] *everyone should undergo for a pregnancy test, which was worth K1,000* [Malawi Kwacha or approximately 1.50 US dollars] *to receive a service. This was for those who wanted to start using family planning (first time).”* – Adult SC

*“I was not prescribed pills though but was rather given condoms because the provider said the only pills available at the hospital were being sold at MK300* [Malawi Kwacha or approximately 0.50 US dollars] *per strip because they belonged to [NGO].”* – Adult SC

In 95% of the visits, at least one of the 11 pre-defined actions related to the four constructs of disrespectful care occurred (Table 3). Three of these actions occurred in over half the visits, lack of privacy (59%), the providers did not ask the client’s preference (57%), and the providers did not mention any other contraceptive methods (56%) (Table 3).

### Differences by case scenario and district

The adult SC who wanted to switch methods received poorer counseling quality compared to the adolescent SC, who was simulating a first-time user. The providers mentioned fewer methods beyond hormonal pills for the adult (31%, CI: 23–40%) compared to the adolescent SC (58%, CI: 48–67%). Also, adult SCs were asked their method preference less (32%, CI: 24–41%) compared to the adolescents (55%, CI: 46–64%). There were no other differences in proportions of adult and adolescent SCs reporting care refusal, long wait times, non-private consultations, or providers advocating for a specific method or exhibiting poor listening/attention. Despite some of the adolescent SCs experiencing humiliating treatment related to their simulated age (Table 3), they experienced the same level of humiliation as the adult SCs (Adult SCs: 18%, CI: 12–26% versus Adolescent SCs: 17%, CI: 11–25%).

The SCs reported refusal of care in three of the six districts. In one of the districts (in the high outcome district group), 58% of SCs were refused service, predominantly because the facilities mandated HTC, in two other districts (one in the high- and one in the low-outcome district group) < 15% of SC visits were refused care, and in three districts none of the SCs were refused care. In the other districts, the SCs noted several instances where the provider encouraged HTC and/or TTV but the SCs were able to decline those services and still receive family planning care (14% of the visits). We found a few statistically significant differences by district, but no clear pattern between high and low outcome district groups (data not shown).

There was no statistically significant difference in the respectful care indicators reported by the SCs during the first half time-period of data collection compared to the second half (data not shown).

## Discussion

We documented important instances of disrespectful care including refusal of services, lack of privacy, poor client-centered care, and instances of non-dignified treatment and humiliation. Since we used simulated clients, we believe this study represents a snapshot of what Malawian woman encounter when accessing FP services. The level of disrespectful care documented in this study has the potential to impact access and utilization.

In 10%^3^ of the consultations, the SC were refused services for not consenting to HIV testing and/or tetanus toxoid vaccination. Malawi policy requires integration of HTC into health care services and the national immunization program recommends TTV for all women of childbearing age, but the policies do not require them as a conditional for accessing family planning services [24, 48]. We found a similar instance in Tanzania where some facilities refused care for antenatal patients because the women did not attend with their husband, an incorrect interpretation of a policy meant to encourage men to attend antenatal care [49]. Some providers may have similarly incorrectly interpreted the policy around TTV and HTC. Because these instances occurred predominantly in one district, it is likely the result of a district-wide decision rather than a national policy or problem.

Half of the SCs who were counseled were counseledin a group setting. Privacy is important particularly for unmarried adolescent clients seeking family planning services [50]. However, group counseling is an effective alternative in resource-constrained areas for imparting accurate contraceptive knowledge but at the expense of privacy [51]. The current Malawi guidelines for health service delivery state that counseling should be conducted privately, however it may not be feasible due to lack of space and human resources [27]. Our sample of facilities ranged from district hospitals to smaller clinics, many of the clinics likely did not have the space and staff to conduct private, individual counseling. Outside of facility-based counseling, innovations such as interactive contraceptive decision-making apps, voice or short message service (SMS) counseling messages, or SMS interactions with FP providers may allow for effective counseling with more privacy and confidentiality [52–54].

In more than half of the consultations, the provider did not counsel the SCs on other method besides hormonal pills and did not directly ask the SCs their method preference. Since the case scenarios involved a first-time user and a client who wanted to switch methods (see Additional file 1), it would have been appropriate to counsel on all FP methods available, specify those available that day, refer for those not available, and ask the woman her preference.^4^ A study on provider perspectives on barriers to reproductive health care for HIV-positive patients in Malawi found the greatest reported barriers to family planning care (in general) was lack of trained staff, resulting in increased caseload and insufficient time for adequate counseling [55]. A facility census of contraceptives stocks in Malawi conducted in 2017 found 62% of facilities had all contraceptives offered on the day of the assessment [28]. It is possible incomplete method counseling was not done intentionally to coerce but instead the providers were limiting their counseling due to stockouts, lack of training, or limited time with clients.

A fifth of the simulated client interactions included humiliating treatment. This finding is similar to other studies in Sub-Saharan Africa [18–20]. About half of Malawian women in a 2017 qualitative study of maternal care (*n* = 30 participants) reported verbal abuse and disrespectful care from health providers [56]. A 2016 survey among women in Tanzania found that 14% reported negative interactions with providers (yelling, scolding or making disparaging comments about the woman) during their most recent visit to a facility for outpatient care, although not specific to family planning [57]. Sudhinaraset et al. developed and validated a person-centered scale for family planning care and excluded verbal and physical abuse due to low correlation with other items in the scale like communication, autonomy, and trust [58]. They hypothesized it was due to low prevalence of abuse in their sample but it could also be indicative that abusive provider behaviors are distinct from other behaviors related to person-centered care. We found limited instances in the published literature on FP clients experiencing verbal or physical sexual harassment from providers or other health staff. A 2015 qualitative study reported concerns of inappropriate touching or comments by family planning providers among Mexican women but no data on how prevalent this physical sexual harassment occurs [59]. This study complements work done by Larson et al. and others, showing that disrespectful care is found in health service sectors outside of labor and delivery and should be considered a system-wide issue [57].

In a 2017 qualitative study in Malawi, youth reported mistrust and expectation of poor treatment due to their age and marital status as an access barrier. In this study we found few differences between the adult and adolescent SCs, and the difference we did find showed the adolescent SCs received better counseling. However, even the low proportion of age-related judgmental comments and humiliating treatment experienced by adolescents may impact their future utilization of family planning services.

### Limitations

This study has some important limitations. First, the client simulation itself may artificially inflate SC’s reporting of disrespectful care. SC training on how to report the details of the consultation may have sensitized them to the idea of disrespectful care, and they may have reported more events that may have otherwise been seen as normal care. A qualitative study on antenatal care showed many Malawian women were not critical of the care they received due to a low expectation of quality [60].

We also recognize the definition of humiliating treatment is subjective and there may be recall bias with SC reports. We did not consider it feasible due to logistical and financial reasons to audio record the SC consultations. The field notes were helpful to elucidate the events reported but having audio-recordings of the consultations would have been ideal to reduce subjectivity and improve accuracy in the assessment of disrespectful care.

We found that the adolescent SC received more complete counseling compared to the adult SC and we cannot determine whether this is due to differences in age/marital status or past contraceptive history. According to Malawi guidelines, the adult SC should have received the same level of counseling as the adolescent SC because she was switching methods and had not used pills “in a long time” (Additional file 1). There may be provider biases against contraceptive use among married women, or it could be the providers are giving more complete counseling to the adolescent because she had no previous contraceptive use. Ideally, we would have used an adult case scenario who was also a first-time user to test this difference and future research may investigate this.

To adhere to ethical standards, the providers were informed that a masked SC would visit them to evaluate their practice. It is possible the providers detected the SC and provided them with higher levels of respectful care than their normal practice, underestimating the prevalence of disrespectful care from this study. However, the SCs reported that neither providers nor health staff appeared to suspect that they were simulated clients. A 1997 study in the United States showed a 3% detection rate of SCs by physicians in two medical centers [61].

We recognize some of the instances described by the SCs may be outlier behaviors and not representative of the health services overall, particularly the case of sexual harassment and the incorrect counseling. However, we felt compelled to document the breadth of experiences that women may encounter when accessing family planning. We also have anecdotal evidence that some humiliating treatment – like being asked for their phone number by health provider – may be higher than the SCs documented in the field notes.

Finally given the parameters of the larger study, we restricted the sample to public sector facilities. So, this study is not generalizable to the private for profit or non-profit sectors (where 6 and 12% of modern contraceptive users source their method, respectively) [22].

### Implications for disrespectful care and health systems

Within the disrespectful care framework, we identified two root causes with different potential solutions. The first was generally poor quality of care due to resource-constrained settings. Poor client centered care (such as inattention or lack of respectful greeting), long waiting times, and facility closures may be due to insufficient staff and facility coverage. Although these issues stem from insufficient human resources and facilities found in many low-income settings, they provide significant barriers preventing clients from accessing the full constellation of family planning methods and may be considered as disrespectful care. Interventions aimed at service quality improvement and health systems strengthening, particularly multi-faceted interventions addressing multiple levels of the health system, may also lead to improvements in respectful care [62, 63]. For instance, a time-motion study of patients in rural Malawi found patients arriving before 10 am had the longest wait times; encouraging patients to arrive after 10 am may alleviate caseload, reduce wait times, and improve satisfaction as long as providers are still available [64]. These interventions should address provider needs – better training, stronger infrastructure, and supports - enabling them to provide higher quality care [65].

The second category is explicit disrespectful and non-dignified care. When looking at these events, we considered facility, provider, and policy-level failures. The refusal of care was likely a policy-level failure in those three districts. Measuring disrespectful care can identify instances where policies are being inappropriately implemented so that programs can intervene and improve. Humiliating treatment likely stems from provider- or facility-level factors. Abuse by providers is thought to result from poor working conditions and disenfranchisement of providers, and also individual level factors such as provider bias or stigma [66, 67]. More research, particularly qualitative studies with providers in under-resourced settings, is needed to determine the root causes of disrespectful care and abuse, and how best to intervene to reduce it. A grievance redress mechanism for clients to report abuse or poor treatment from providers can allow supervisory follow-up or disciplinary action if necessary and is a critical component of health system accountability [68]. We noted disrespectful care can occur throughout the health system but the humiliating treatment documented by some of the adolescent SCs is specific to family planning. The public disclosure of adolescent or young, unmarried women’s sexual activity that occurs when they access family planning services makes them vulnerable to exploitation or harassment.

## Conclusion

This study quantifies and documents disrespectful care provided through public sector facilities, where 80% of modern contraceptive users in Malawi receive contraceptives [22]. We found important instances of disrespectful care, most related to low quality readiness (i.e. poor training, limited space for client privacy, and burdened health workforce) and several instances of maltreatment of clients. While we studied this issue in Malawi, these findings may be applicable in other settings in both low- and high-income countries, given the ubiquitous reports of disrespectful care and abuse in maternal care [15].

First, continued effort to improve quality of care with an emphasis on client treatment at the policy, health system, facility, and provider levels will support respectful care. Second, governments and programs require regular quality assessments that include evaluation and reporting instances of disrespectful care and abuse. Facility assessment tools may be revised to include this information. Counseling completeness is already incorporated into the Service Provision Assessment (SPA) protocol for direct observation of health services [69]. A relatively small set of simple questions asking about service refusal or humiliating treatment by providers/health staff could be asked during client exit interviews in the SPA or other health facility assessments. The literature shows women are reporting this abusive or humiliating care when accessing health services, but cognitive interviewing or other techniques are required on how to best capture their experiences using a survey to measure prevalence. Finally, more work is needed to identify and implement interventions or best practices to reduce disrespectful care of clients. Globally, respectful, person-centered care should be prioritized for family planning and all health programs.

## Supplementary Information

**Additional file 1.** Description of two case scenarios adopted by the simulated clients.

## Data Availability

The datasets used and/or analyzed during the current study are available from the corresponding author on reasonable request.

## Abbreviations

DHS: Demographic and Health Survey
FP: Family planning
HTC: HIV testing and counseling
LMICs: Low-and-middle-income countries
SMS: Short message service
SC: Simulated client
TTV: Tetanus Toxoid vaccination

## Footnotes

1 Personal communication, Amani Siyam, World Health Organization Quality of Care indicators, Draft. April 2017

2 Personal communication, Patrick Msukwa, Field Coordinator, Wadonda Consult Limited, 19th December 2019

3 Excluding the 1.8% of the consultations that were refused because the facility was closed during operating hours.

4 Personal communication, John Chawawa, Family Planning Coordinator and Nursing Officer, Zomba district health office, Reproductive health Directorate - Ministry of Health Malawi. 7th January 2020
