# Supplementary material for: Disrespectful care in family planning services among youth and adult simulated clients in public sector facilities in Malawi
Source: BMC Health Serv Res. 2021 Apr 14;21:336. doi: 10.1186/s12913-021-06353-z (PMC8045277; doi:10.1186/s12913-021-06353-z)
Supplement: Supplementary file 1 — Additional file 1. Description of two case scenarios adopted by the simulated clients. [file 12913_2021_6353_MOESM1_ESM.docx]

Additional file

Additional file 1: Description of two case scenarios adopted by the simulated clients.

| **Adult, married woman who is switching methods** |
| --- |
| **Background**  She began using Depo-Provera for the first time 6 weeks after the birth of her youngest child. She has heavy menstrual bleeding for the past six months. She went to clinic and got pills to treat it,but the heavy bleeding continues. She went back to the clinic and got different pills to treat it, but the bleeding continues. She would like to stop taking the injections and change to a different method.  **Partner information**  She is married. Her husband is not supportive of her contraceptive use and wants more children.  **Parity and fertility preference**  She had three live birth and three living children. Her youngest child was born seven and half months ago. She does not want more children  **Medical history**  She is sexually active and not sexually active outside her marriage. And believes that she has no risk for sexually transmitted infections but has not been tested. She has no pregnancy symptoms. Her last period was 1 week ago, and her cycle is usually regular. She is still breastfeeding some but has introduced solids. She had never smoked. Otherwise she is healthy.  **Preference**  She does not want to have an implant because her sister became pregnant due to a faulty implant. She is interested in learning about intrauterine devices but concerned about the side effects. She would like to take pills because she took them a long time ago and had no side effects. |
| **Adolescent, unmarried woman who is a first-time user** |
| **Background**  The client is a 17-year-old girl who wants to begin contraceptives.  **Partner information**  She and her boyfriend recently became sexually active. Her boyfriend does not like to use male condoms and neither really know how to use them.  **Parity and fertility preference**  She has no prior pregnancies. She would like to have children but wants to wait until she has completed school. She is afraid of getting pregnant.  **Medical history**  She is unmarried but sexually active with one partner (boyfriend). She has never used any form of birth control. She has no symptoms of pregnancy. Her last period was 1 week ago, and her cycle is regular. She is not sexually active outside her relationship. She does not know her STI status and has never been tested. She does not smoke and is otherwise healthy.  **Preference**  Oral contraceptives: Several of her friends are using oral contraceptives, and they have notgotten pregnant yet, even though they sometimes forget to take the pills. She thinks pills would be good for her too, but she is worried about forgetting to take the pills. She is also nervous about her parents finding her pills and knowing she is sexually active. |
